# Supplementary material for: A leader-follower model for discrete competitive facility location problem under the partially proportional rule with a threshold
Source: PLoS One. 2019 Dec 3;14(12):e0225693. doi: 10.1371/journal.pone.0225693 (PMC6890218; doi:10.1371/journal.pone.0225693)
Supplement: Attachment — Submitted filename: PONE-D-19-21394_comments.pdf [file pone.0225693.s002.pdf]

Reviewer's comments about PONE-D-19-21394

This paper is about a new method for modeling the behavior of customers when they cope with a competing market for an exclusive service. To the best of my knowledge, the partially proportional rule with a threshold is not considered in the literature before. I think that the proposed practical idea is novel and therefore, the manuscript deserves publishing.

Major comments

1. First paragraph of Section 2: The attraction of a firm should be defined as the sum of attraction of its facilities; otherwise, the reader would be confused.
2. The numbers under the partially proportional rule with threshold $\delta = 1$ should be modified. They are not described, but I think they are probabilities of selection. However, they are misleading, since the reader thinks that it is possible that a selection is made between a yellow and a blue facility. This is not possible according to the text. I think you should separate the probability of selection of the firm and the probability of selecting a facility, and the multiplication is misleading.

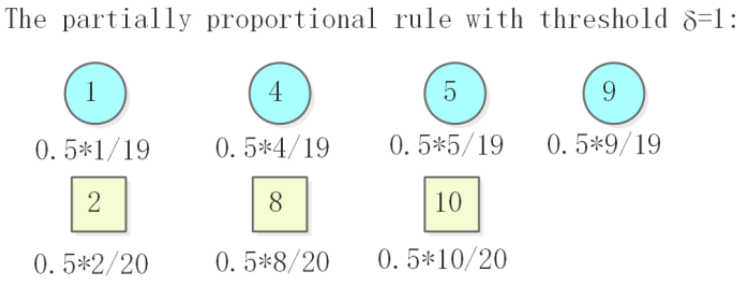

3. What do the brackets mean in Equation (1)? If you do not mean the integral part, please use parentheses.

Minor comments

1. The sentence “if the total attraction of two firms are equal or approximated”, does not have correct meaning that you want.
2. The grammatical errors in the manuscript should be corrected. Some of them are as follows.
    a. Competition type usually be divided into two categories
    b. will not take actions against new enter firm
    c. Section 4 illustrates the effectiveness of the algorithm through numeral tests (it should be numerical tests)
    d. to the exclusive of these services (exclusiveness)
